# Suspected Horse-to-Human Transmission of MRSA ST398

**DOI:** 10.3201/eid1706.101330

**Published:** 2011-06

**Authors:** Engeline van Duijkeren, Lenny ten Horn, Jaap A. Wagenaar, Marco de Bruijn, Laura Laarhoven, Koen Verstappen, Willemien de Weerd, Nico Meessen, Birgitta Duim

**Affiliations:** Author affiliations: Utrecht University, Utrecht, the Netherlands (E. van Duijkeren, J.A. Wagenaar, L. Laarhoven, K. Verstappen, B. Duim);; University of Groningen, Groningen, the Netherlands (L. ten Horn, N. Meessen);; Wolvega Equine Hospital, Oldeholtpade, the Netherlands (M. de Bruijn, W. de Weerd)

**Keywords:** MRSA, methicillin-resistant Staphylococcus aureus, transmission, horse, human, infection, bacteria, genotyping, letter

**To the Editor**: Methicillin-resistant *Staphylococcus aureus* (MRSA) is spreading worldwide among humans and animals, including horses. Many reports of MRSA colonization and infection in horses come from Canada and involve MRSA of sequence type (ST) 8, classified by pulsed-field gel electrophoresis (PFGE) as Canadian MRSA-5 or USA500. ST8 is thought to be a human epidemic clone that has adapted to horses (*1*). Another MRSA type, ST398, has recently begun spreading in Europe and North America and is associated with livestock (*2*). In the Netherlands, MRSA of ST8 (*spa*-type t064) and ST398 (*spa*-type t011), which belong to the livestock–associated CC398, predominate in clinical samples from horses (*3*). To date, human clinical infections with livestock–associated MRSA are uncommon in persons who have not had contact with pigs or calves (*2*). In this case study, we describe the suspected transmission of MRSA ST398 between a horse and a girl, which resulted in infection of the girl’s right foot.

In the Netherlands, a 16-year-old girl with spinal muscular atrophy type II (wheelchair-bound and needing artificial ventilation) sought treatment at a hospital for an infected wound on her right foot thought to be caused by an insect bite (online Appendix Figure, www.cdc.gov/EID/content/17/6/1137-appF.htm). The girl was treated as an outpatient. The infection did not respond to empirical treatment with clindamycin and ciprofloxacin. From the infected wound, a MRSA strain that was resistant to clindamycin, ciprofloxacin, erythromycin, gentamicin, kanamycin, tetracycline, and trimethoprim/sulfonamide, and susceptible to rifampin and fusidic acid, was isolated 39 days after initial treatment. Identification of the bacteria and susceptibility testing were performed by using Vitek 2 (bioMérieux, Marcy l’Etoile, France). The girl did not have a history of hospital admission in other countries, nor contact with pigs or calves, but had had intensive contact with a foal. No information was available about hand hygiene practices the girl used after stroking the foal.

Because the girl was a frequent visitor to the hospital, according to the national hospital MRSA guidelines, decolonization therapy was indicated. Before therapy began, her 3 household members and their animals (7 adult Friesian horses, 2 dogs, and 2 cats) were screened for MRSA by enrichment culturing. Nasal swabs were taken from the animals; nasal, throat, and perineal samples were taken from the humans. MRSA with an identical susceptibility pattern was isolated from a sample taken from the nares of the girl’s healthy Friesian foal. The foal had been hospitalized at a horse clinic 2 months earlier because of a wound infection and had been treated with antimicrobial drugs, but no samples had been taken from the horse’s wound at that time. All other screening samples were negative for MRSA. The girl’s wound healed after application of mupirocin ointment to the nares and perineum (3×/d for 5 days), washing of the body with chlorhexidine shampoo (1×/d for 5 days), and oral administration of fusidic acid and rifampin for 7 days; samples taken were negative for MRSA. The girl was advised not to touch the foal until it too was negative for MRSA. Without therapy, and within 3 months, the foal was negative for MRSA (confirmed by 3 repeated negative cultures of nasal samples by enrichment culturing).

Isolates from the girl and the horse were further investigated by Martineau PCR targeting the *tuf* gene (*4*), *mecA* PCR (*5*), ST398-specific PCR (*6*), *spa* typing (*7*), and PFGE using *Sma*I and *Cfr*9I as restriction enzymes (*8*). Both isolates were identified as *S. aureus*, were *mecA* positive, belonged to ST398, were *spa* type t011, were nontypeable by PFGE using *Sma*I, and had indistinguishable PFGE patterns using *Cfr*9I.

Colonization of persons in contact with infected or colonized horses has been widely reported (*1*–*3*). Clinical MRSA infections of humans associated with horse contact, however, are rare and, to our knowledge, only 2 reports have been published. The first report of a human infection came from Canada and concerned a veterinarian who had a tattoo site infection with Canadian MRSA-5, (ST8, SCC*mec* type IV, *spa* type t007) (*9*). Human skin infections with Canadian MRSA-5 associated with horse contact were also reported from 3 persons who worked in a foal nursery (*10*). MRSA ST398 *spa*-type t011 are cultured regularly from equine samples at the horse clinic (*3*); therefore, the foal probably became colonized during its hospitalization. Livestock-associated MRSA infections are rare in humans in the region where the girl lives, and human-to-human transmission of MRSA ST398 is uncommon. In addition, the girl was severely handicapped and could not travel freely. Therefore, we theorize that the foal, which was stabled in a barn at her home, was the most likely source of the infection. It is also possible that the girl and the foal contracted MRSA from an unidentified common source or that the foal was exposed by the girl, although this is less likely. Close collaboration between the pediatrician, infection control practitioner, veterinarians, and the human microbiologist was necessary to identify the suspected source of infection.

## Figures and Tables

**Appendix:**
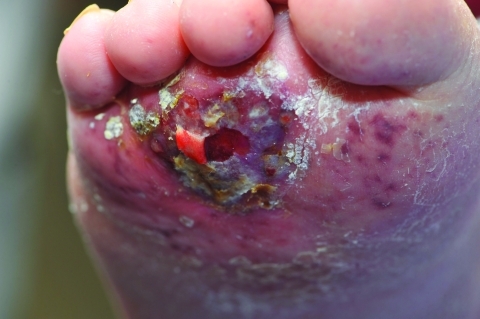
Figure. Infected wound, initially thought to be caused by an insect bite, on the right foot of a 16-year-old girl who was wheelchair-bound because of spinal muscular atrophy type II, the Netherlands.
